# Time-order-errors and duration ranges in the Episodic Temporal Generalization task

**DOI:** 10.1038/s41598-017-02386-9

**Published:** 2017-06-01

**Authors:** Ezequiel Mikulan, Manuel Bruzzone, Manuel Serodio, Mariano Sigman, Tristán Bekinschtein, Adolfo M. García, Lucas Sedeño, Agustín Ibáñez

**Affiliations:** 1 Laboratory of Experimental Psychology and Neuroscience (LPEN), Institute of Cognitive and Translational Neuroscience (INCYT), INECO Foundation, Favaloro University, Buenos Aires, Argentina; 2 National Scientific and Technical Research Council (CONICET), Buenos Aires, Argentina; 3 Di Tella University, Buenos Aires, Argentina; 4 Consciousness and Cognition Lab, Department of Psychology, University of Cambridge, Cambridge, UK; 5 Faculty of Education, National University of Cuyo (UNCuyo), Mendoza, Argentina; 6 Universidad Autónoma del Caribe, Barranquilla, Colombia; 7 Center for Social and Cognitive Neuroscience (CSCN), School of Psychology, Universidad Adolfo Ibañez, Santiago de Chile, Chile; 8 Australian Research Council Centre of Excellence in Cognition and its Disorders, Sydney, Australia

## Abstract

The current model of the Episodic Temporal Generalization task, where subjects have to judge whether pairs of auditory stimuli are equal in duration, predicts that results are scale-free and unaffected by the presentation order of the stimuli. To test these predictions, we conducted three experiments assessing sub- and supra-second standards and taking presentation order into account. Proportions were spaced linearly in Experiments 1 and 2 and logarithmically in Experiment 3. Critically, we found effects of duration range and presentation order with both spacing schemes. Our results constitute the first report of presentation order effects in the Episodic Temporal Generalization task and demonstrate that future studies should always consider duration range, number of trials and presentation order as crucial factors modulating performance.

## Introduction

Time has been a matter of ardent debate across many disciplines^1^. Within neuroscience, not only does it constitute an important topic in its own right^2^, but it also impinges on the field’s fundamental areas of inquiry, including consciousness^3^, motor control^4^, memory^5^, artificial intelligence^6^, and neural dynamics^7^. Likewise, the study of timing abnormalities is pivotal for research on pathologies such as Parkinson’s disease and schizophrenia^8^. In brief, understanding timing, time perception, and their neural basis proves fundamental for contemporary neuroscience^9^.

Even though many relevant models and theories have been proposed^10^, some basic questions are still unsolved. Here we aimed to address one of them: *is timing equal across different scales*?

Multiple studies have addressed this issue based on a distinction between sub- and supra-second durations^11^, with contradictory results. Some studies show that response variability increases linearly as a function of duration, thus following Weber’s Law, in a range that goes from a few hundred milliseconds to a few seconds –see ref. 12 for a systematic investigation. However, other reports indicate that this linear property stops holding at some point between 1 and 2 seconds –see ref. 13 for a review.

This controversy is epitomized by the antinomy between two major conceptual frameworks used to account for timing mechanisms in the brain: the “common timing hypothesis” and the “distinct timing hypothesis”^14^. Whereas the former assumes a single timing mechanism irrespective of duration, the latter posits dissociable mechanisms for sub- and supra-second durations.

A typical approach to study time perception is to have participants judge whether two durations are equal^15^. This so-called Temporal Generalization task has two main versions for humans. In the original one^16^, participants learn a standard duration at the beginning of the experiment and are then presented with several to-be-compared durations. Instead, in the *Episodic* version^17^, subjects judge the durations of two successive stimuli on a trial-by-trial basis. Stimuli are constructed in a similar way for both versions: a set of comparison durations is generated multiplying a standard duration (e.g., 400 ms, or values from a range such as 300 to 500 ms) by a series of ratios (e.g., 0.5, 1, 1.5). In the original version, a clear standard is learnt at the beginning and participants judge whether it is equal to each of the following durations. In the *Episodic* version, presentation order is counterbalanced so that in half of the trials the standard comes first and in the other half it comes second.

Both tasks showed a similar pattern of results with sub-second durations: the obtained psychometric functions were asymmetrical, with a higher proportion of “equal” responses on the right tail, that is, when the ratio was higher than 1^16, 17^. The same pattern was found using the original standard version, with durations ranging from 2 up to 8 seconds^18^. Importantly, results were superimposed between duration ranges when plotted in a relative scale, that is, as a function of the proportion between standard and comparison durations. This was interpreted as confirmation of the common timing hypothesis. However, no studies have yet used supra-second durations as standards in the *Episodic* version.

Traditionally, temporal generalization results have been interpreted within Scalar Expectancy Theory, which, in brief, states that durations are estimated via accumulation of pulses. Within this framework, in the temporal generalization task subjects would compare the two values of the estimated durations and then decide based on their normalized absolute difference^19^. Therefore, according to this model, results should not be affected by presentation order of the stimuli, something known as the “balance condition”^20^, nor by duration range. Presentation order effects have been shown in a wide variety of time perception tasks, termed “time-order-errors” (TOE) within this context (see ref. 21 for a review), but never in the Episodic Temporal Generalization (ETG) task. In fact, no previous studies using the task tested for any effect of this kind^17, 22–24^.

In addition, evaluating symmetry with linearly spaced proportions (i.e. 0.25, 0.50, 0.75, 1, 1.25, 1.50, 1.75), as is usually the case with the ETG task^17, 19, 22^, implies an unbalanced comparison. Symmetry within this setting would indicate that a similar amount of “equal” responses were obtained when comparing, for example, ratios 0.25–1 (1:4) and 1.75–1 (1.75:1) or vice versa (4:1 and 1:1.75). A more meaningful comparison would arise from using logarithmically spaced proportions, so that, following the example above, ratios, 0.25–1 (1:4) and 4–1 (4:1) could be contrasted.

Moreover, the property of superposition has been tested via visual inspection or ANOVAs^17, 19^, none of which is robust to such an end. The first one proves inadequate because it does not establish a decisional boundary to accept or reject hypotheses, and the second because it can produce spurious results when used with proportional data^25, 26^. A more convenient approach would be to compare Weber Fractions (WF) between duration ranges and test whether they remain constant, in which case a scalar relationship could be assumed to exist between them.

Against this background, our study pursued three main objectives. First, we tested the prediction, derived from the traditional ETG model, that presentation order had no effect on performance. Second, we examined the symmetry/asymmetry of the temporal generalization gradients taking presentation order into account and using linear and logarithmically spaced proportions, so that symmetry could be properly assessed. Third, we compared WFs of sub- and supra-second ranges to test their compliance with the scalar property of timing.

To address these aims, we conducted three experiments. Experiment 1 was designed with a number of trials similar to that of previous studies using the task^17, 22^ and comprised linearly spaced proportions. As taking presentation order into account reduced the number of trials of each ratio to a half, we conducted Experiment 2, in which the same task was administered but with a threefold increase in trials. Experiment 3 had the same number of trials as Experiment 2 but proportions were logarithmically spaced. With this combination of experiments, we aimed to address some critical gaps in the ETG framework.

## Method

### Participants

Eighteen subjects participated in Experiment 1 (F = 10; $\overline{{\rm{x}}}$ age = 24.22; *s* age = 3.12; $\overline{{\rm{x}}}$ years of education = 17.78; *s* years of education = 3.83), 20 in Experiment 2 (F = 10; $\overline{{\rm{x}}}$ age = 25.47; *s* age = 3.94; $\overline{{\rm{x}}}$ years of education = 18.87; *s* years of education = 3.04), and 18 in Experiment 3 (F = 11; $\overline{{\rm{x}}}$ age = 24.39; *s* age = 3.53; $\overline{{\rm{x}}}$ years of education = 18.94; *s* years of education = 2.82), after signing informed consent. Subjects participated in only one of the experiments. All of them reported normal hearing, right-handedness, and absence of neurological and psychiatric antecedents, and they were naïve as to the purpose of the study. The experiments were approved by the local ethics committee (INECO Foundation) and were conducted in accordance with the Declaration of Helsinki.

### Stimuli

Auditory stimuli were 500-Hz tones created and delivered using Matlab (Mathworks Inc.) and Psychtoolbox^27^ through Sennheiser HD202 headphones at 65db on a MacBook Pro notebook. Before the experiment, the software created 7 blocks of 16 trials; half of the stimuli corresponded to the *Sub-Second* condition and the other half to the *Supra-Second* one. For each trial, a *Standard* (S) duration was selected from a uniform distribution, ranging from 300 to 500 ms in the *Sub-Second* condition, and from 1200 to 2000 ms in the *Supra-Second* one. Then, S was multiplied by one of 7 ratios (linearly spaced in Experiments 1 and 2: 0.25, 0.50, 0.75, 1.0, 1.25, 1,50, 1.75; logarithmically spaced in Experiment 3: 0.25, 0.40, 0.63, 1, 1.59, 2.52, 4) to create a *Comparison* (C) duration, so that on each block there was one trial of each ratio for each condition (i.e., 7 ratios × 2 conditions = 14 trials). An additional 1.0 ratio trial was then added to each condition. Finally, the order of the trials was randomized and each trial was assigned a random counterbalanced presentation order so that on half of the trials the S was presented first, and on the other half the first stimulus presented was C. In Experiment 1 a total of 112 trials were obtained from each subject in an average of 16 minutes (σ = 36 s). In Experiment 2, the task was repeated three times with 5 minute breaks between them, therefore 336 trials were obtained from each subject, in 62 minutes on average (σ = 9 m). In Experiment 3, 336 trials were obtained from each subject in 68 minutes on average (σ = 5 m). See Supplementary Figures 1, 2, and 3 (S1, S2, and S3) for further details.

### Procedure

**Figure 1:**
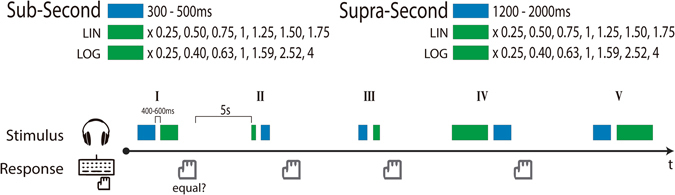
Diagram of the experimental paradigm. Blue rectangles represent *Standard* durations and green rectangles represent *Comparison* durations. Linearly spaced proportions (LIN) were used in Experiments 1 and 2 and logarithmically spaced proportions (LOG) in Experiment 3.

Participants were informed that they would hear sequences of two tones and that their task was to decide whether both sounds had the same duration (Fig. 1). Each trial started with a 5-s inter-trial interval that was followed by the presentation of a tone, a gap (randomly chosen from a uniform distribution from 400 to 600 ms), and a second tone. Participants had to respond with their right hand on the notebook keyboard. To indicate that the tones were equal, they had to press the down arrow key with their index finger; to indicate that they were not, they had to press the right arrow with their middle finger. Importantly, post-task debriefing showed that although all participants detected two duration ranges, none of them realized that there were standard and comparison distributions.

### Data Analysis

Statistical analyses were performed on R software^28^. All subjects were included in them. Following previous reports^18^, we plotted the temporal generalization gradients as a function of comparison durations and tested their asymmetry by comparing the proportion of “equal” responses on the three Ratios below 1 (C-Shorter), against the proportion of “equal” responses on the three Ratios above it (C-Longer) using Wilcoxon Signed-Rank tests. Statistical results were corrected for multiple comparisons using the Holm-Bonferroni method.

In order to characterize the TOE, we first estimated the point of subjective equality (PSE) using the smoothing spline curve-fitting method from Matlab’s (Mathworks Inc.) Curve Fitting Toolbox (with automatic selection of the smoothing parameter), and finding the maximum of the resulting curve. We then defined the TOE following Fechner’s definition of constant error (CE)^21^:

1 $$CE=PSE-st$$

where PSE denotes the point of subjective equality and *st* the standard duration. Within this context, the sign of CE denotes the sign of the TOE when the standard is presented first. When the standard was presented in second place, the sign of the TOE was computed as *st* – PSE. We report the magnitude of the TOE as a percentage of the standard (%TOE)^29^. %TOEs were submitted to a rm-ANOVA with standard duration (Sub-second/Supra-second) and order (S-C/C-S) as factors.

Weber Fractions (WF) were calculated as:

2 $$WF=DL/PSE$$

where DL denotes the difference limen and PSE the point of subjective equality^30^. Within this analysis, the PSE and DL were calculated as the mean and standard deviation of a fitted Gaussian function, respectively. WFs were submitted to a rm-ANOVA with standard duration (Sub-second/Supra-second) and order (S-C/C-S) as factors. To control that the PSEs did not differ between methods we compared them using a Wilcoxon Signed-Rank test.

Effect sizes in all cases were calculated via generalized eta squared (η^2^_G_)^31^; these were considered as small if η^2^_G_ = 0.02, medium if η^2^_G_ = 0.13, and large if η^2^_G_ = 0.26. Holm-Bonferroni corrected *post hoc* t-tests were used for pairwise comparisons.

## Results

### Experiment 1

**Figure 2:**
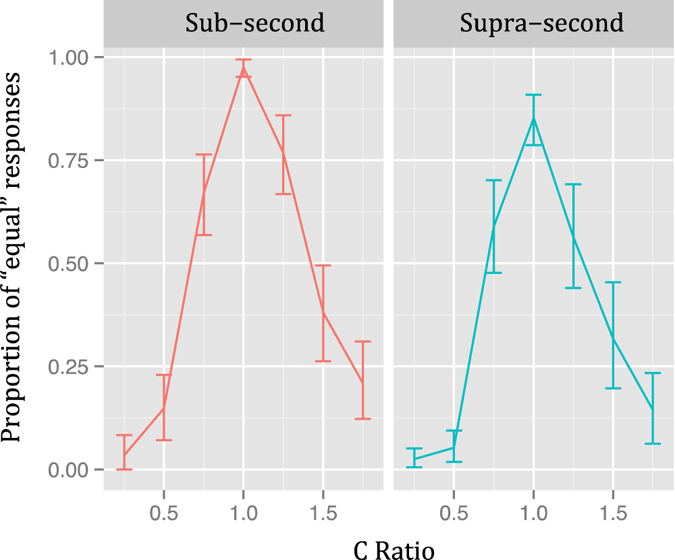
Temporal generalization gradients of Experiment 1 (linearly spaced ratios). Proportion of “equal” responses as a function of the ratio of the comparison duration when collapsing presentation orders Vertical lines represent 95% confidence levels.

The proportion of “equal” responses (PE) for each ratio and duration, when collapsing presentation orders, is shown in Fig. 2. Even though visual inspection suggests that both temporal generalization gradients were asymmetrical, Wilcoxon tests proved that this was significant only in the *Sub-Second* condition (V = 135.5, *p* < 0.01), where a higher PE was found when C-Ratio > 1. In the *Supra-Second* condition, the difference was not significant (V = 220, *p* = 0.12).

**Figure 3:**
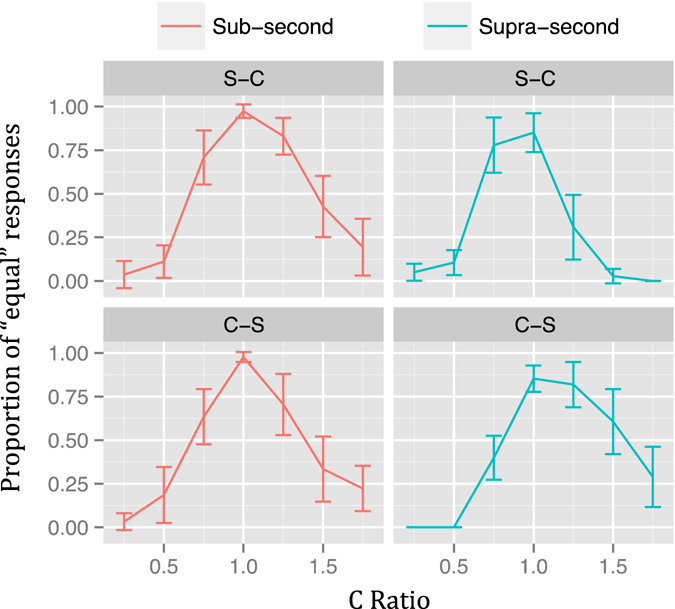
Temporal generalization gradients of Experiment 1 (linearly spaced ratios) by presentation order. Proportion of “equal” responses for the Sub-Second (left) and Supra-Second (right) conditions by presentation order. Vertical lines represent 95% confidence levels.

The PE for each ratio and duration when including presentation order as an additional variable is shown in Fig. 3. Visual inspection again suggests that all temporal generalization gradients were asymmetrical, which, in this case, was true for all comparisons. In the *Sub-Second* condition, both presentations orders, S-C (V = 30, *p* < 0.05) and C-S (V = 40, *p* < 0.05) had a greater PE when C-Ratio > 1. In the *Supra-Second* condition, the C-S presentation order also had a greater PE when C-Ratio > 1 (V = 139, *p* < 0.01). Interestingly, in the S-C order of the *Supra-Second* condition the asymmetry was in the opposite direction, that is, the PE was higher when C-Ratio < 1 (V = 1, *p* < 0.01). Visual inspection also suggests that the temporal generalization gradients of the *Supra-second* condition are shifted to the left and right in the S-C and C-S orders, respectively.

This part of the analysis revealed that the asymmetries of temporal generalization gradients were not equal between the two duration ranges considered. Moreover, when presentation order of the stimuli was taken into account, results suggested that this occurred because of a shift of the temporal generalization gradients in the *Supra-Second* condition that depended on the presentation order of the stimuli (Fig. 3). The presentation order effect in combination with the asymmetrical gradients (the latter probably due to the linear spacing of the comparison proportions) undermined the comparison of WFs, as they rely on the estimation of the spread of the temporal generalization gradients. This analysis was therefore not carried out for this Experiment.

In Experiment 1, we used a similar amount of trials than previous studies that employed the task^17, 22^. Partitioning trials by presentation order left approximately 3–4 trials per ratio and order combination for each subject, which might have led to inaccurate results. In order to overcome this limitation we replicated Experiment 1 but this time we collected three times more trials per subject.

### Experiment 2

**Figure 4:**
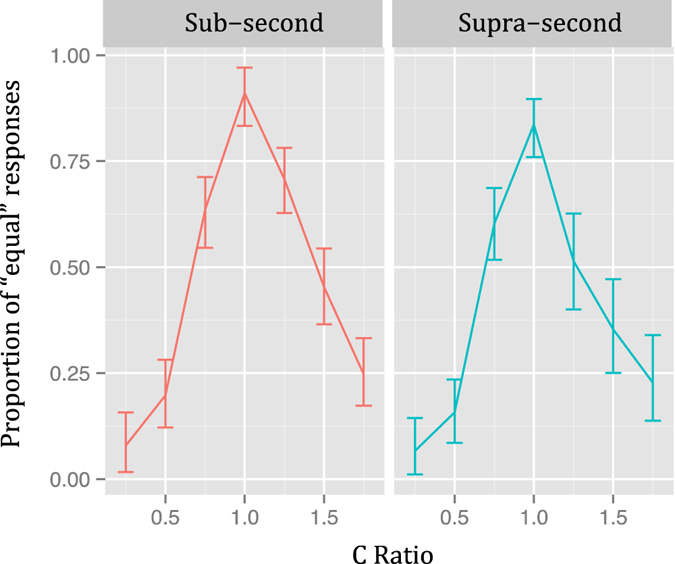
Temporal generalization gradients of Experiment 2 (linearly spaced ratios). Proportion of “equal” responses as a function of the ratio of the comparison duration when collapsing presentation orders Vertical lines represent 95% confidence levels.

The PE for each ratio and duration, when collapsing presentation orders, is shown in Fig. 4. Visual inspection again suggests that both temporal generalization gradients were asymmetrical, and again Wilcoxon tests proved that this was significant only in the *Sub-Second* condition (V = 89, *p* < 0.001), where a higher PE was found when C-Ratio > 1. In the *Supra-Second* condition, the difference was not significant (V = 282, *p* = 0.17).

**Figure 5:**
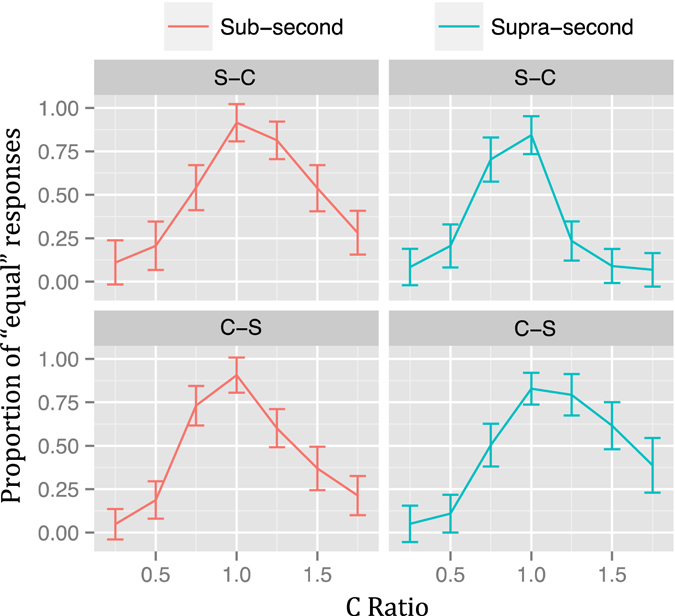
Temporal generalization gradients of Experiment 2 (linearly spaced ratios) by presentation order. Proportion of “equal” responses for the Sub-Second (left) and Supra-Second (right) conditions by presentation order. Vertical lines represent 95% confidence levels.

The PE for each ratio and duration when including presentation order as an additional variable is shown in Fig. 5. Visual inspection once more suggests that all temporal generalization gradients were asymmetrical, which, in this case, was true for all comparisons but one. In the *Sub-Second* condition, the S-C presentation order had a higher PE when C-Ratio > 1 (V = 1, *p* < 0.001) and in the C-S order the difference was not significant (V = 76, *p* = 0.29). As in Experiment 1, in the *Supra-Second* condition, the C-S presentation order had a greater PE when C-Ratio > 1 (V = 12, *p* < 0.001) and in the S-C order the asymmetry was in the opposite direction, that is, the PE was higher when C-Ratio < 1 (V = 196, *p* < 0.01). Visual inspection also suggests that temporal generalization gradients are shifted but this time also in the *Sub-second* range. Interestingly, the directions of the shifts seem to be inverted between duration ranges and orders.

This part of the analysis revealed that, as in Experiment 1, the asymmetries of the temporal generalization gradients were not equal between the duration ranges considered in the study. Experiment 2 showed that when collecting more trials per subject, the asymmetry of the C-S order of the Sub-second condition was not significant, in contrast with Experiment 1. Furthermore, Experiment 2 suggests that the shifts of the temporal generalization gradients that were observed in the *Supra-second* condition also appeared in the *Sub-second* range but with opposite sign.

**Figure 6:**
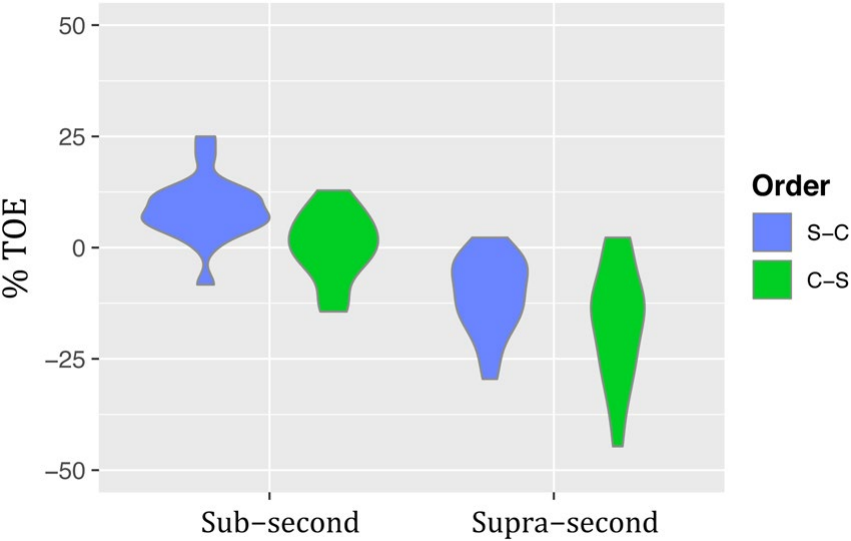
Time-order-errors. Violin plots of the magnitude and sign of the time-order-errors for each duration range and presentation order, as percentage of standard duration, across participants of Experiment 2 (linearly spaced ratios). The corresponding ANOVA showed a main effect of duration range (*p* < 0.001, η^2^_G_ = 0.48), a main effect of presentation order (*p* < 0.001, η^2^_G_ = 0.14) and a non-significant interaction (*p* = 0.45, η^2^_G_ = 0.009).

In order to characterize the presentation order effect (TOE) we computed the %TOE of each subject of Experiment 2 (Fig. 6) and conducted a rm-ANOVA. It showed a main effect of Duration (*F*_1,19_ = 93.15, *p* < 0.001, η^2^_G_ = 0.48), with a higher %TOE in the *Sub-second* condition; a main effect of Order (*F*_1,19_ = 27.36, *p* < 0.001, η^2^_G_ = 0.14), with a higher %TOE in the S-C order; and a non-significant Duration x Order interaction (*F*_1,19_ = 0.58, *p* = 0.45, η^2^_G_ = 0.009). The %TOE was positive in the *Sub-second* range ($\overline{{\rm{x}}}$ = 5.68) and negative in the *Supra-second* range ($\overline{{\rm{x}}}$ = −13.79).

Again, the combination of the different presentation order effects and the asymmetrical gradients hindered the comparison of WFs, so no such analysis was conducted. The linear spacing of the comparison proportions probably caused the asymmetrical gradients. We therefore conducted a third experiment, this time with logarithmically spaced ratios, so that the resulting gradients presumably became more symmetrical and allowed a meaningful comparison of WFs.

### Experiment 3

**Figure 7:**
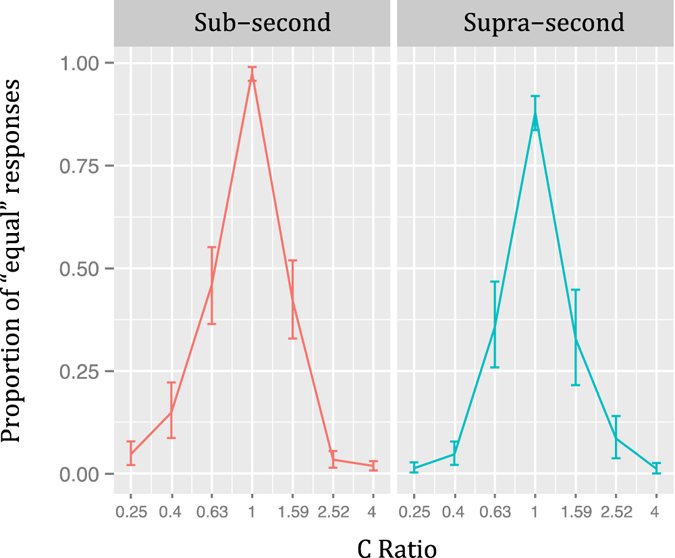
Temporal generalization gradients of Experiment 3 (logarithmically spaced ratios). Proportion of “equal” responses as a function of the ratio of the comparison duration when collapsing presentation orders Vertical lines represent 95% confidence levels.

The PE when collapsing presentation order is shown in Fig. 7. Here, visual inspection suggests that temporal generalization gradients were not asymmetrical, which Wilcoxon tests proved to be correct (Sub-second: V = 417.5, *p* = 0.28; Supra-second: V = 274, *p* = 1).

**Figure 8:**
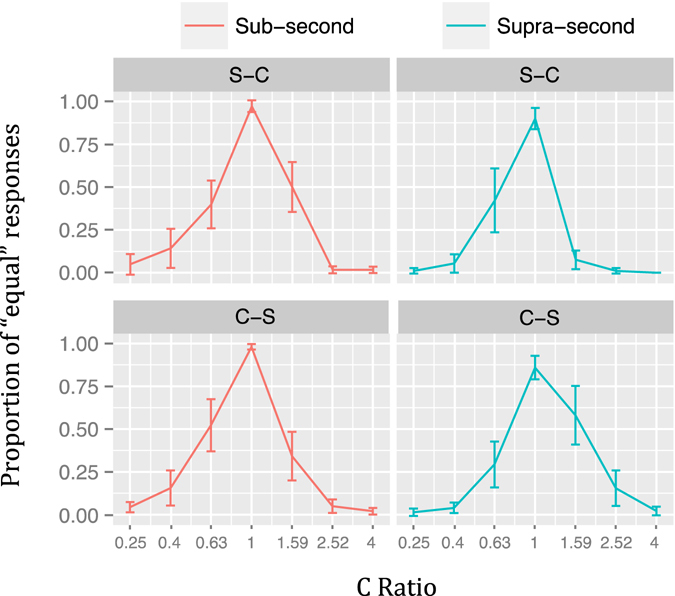
Temporal generalization gradients of Experiment 3 (logarithmically spaced ratios) by presentation order. Proportion of “equal” responses for the Sub-Second (left) and Supra-Second (right) conditions by presentation order. Vertical lines represent 95% confidence levels.

The PE for each ratio and duration when including presentation order as an additional variable is shown in Fig. 8. Visual inspection suggests that all temporal generalization gradients were symmetrical, which was true for all comparisons but one. The only significantly asymmetrical gradient was found in the *Supra-second* S-C condition with a higher PE when C-Ratio < 1 (V = 108, *p* < 0.05). All remaining comparisons were not significant (Sub-second S-C: V = 66, *p* = 1; Sub-second C-S: V = 144.5, *p* = 0.54; Supra-second C-S: V = 32, *p* = 0.08).

**Figure 9:**
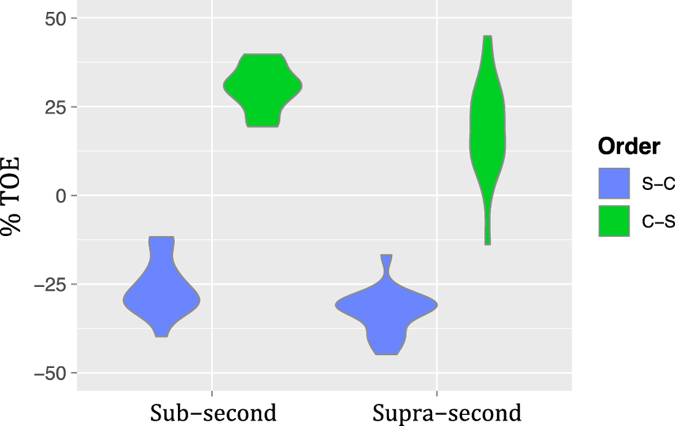
Time-order-errors. Violin plots of the magnitude and sign of the time-order-errors for each duration range and presentation order, as percentage of standard duration, across participants of Experiment 3 (logarithmically spaced ratios). The corresponding ANOVA showed a main effect of duration range (*p* < 0.001, η^2^_G_ = 0.21), a main effect of presentation order (*p* < 0.001, η^2^_G_ = 0.90) and a non-significant interaction (*p* = 0.056, η^2^_G_ = 0.01).

The TOE analysis from Experiment 3 (Fig. 9) showed a main effect of Order (*F*_1,17_ = 2344.41, *p* < 0.001, η^2^_G_ = 0.90), with a higher %TOE in the C-S order; a main effect of Duration (*F*_1,17_ = 16.00, *p* < 0.001, η^2^_G_ = 0.21), with a higher %TOE in the *Sub-second* condition; and a non-significant Duration x Order interaction (*F*_1,17_ = 4.20, *p* = 0.056, η^2^_G_ = 0.01). The %TOE was negative in the C-S order ($\overline{{\rm{x}}}$ = −29.38) and positive in the S-C order ($\overline{{\rm{x}}}$ = 24.42).

**Figure 10:**
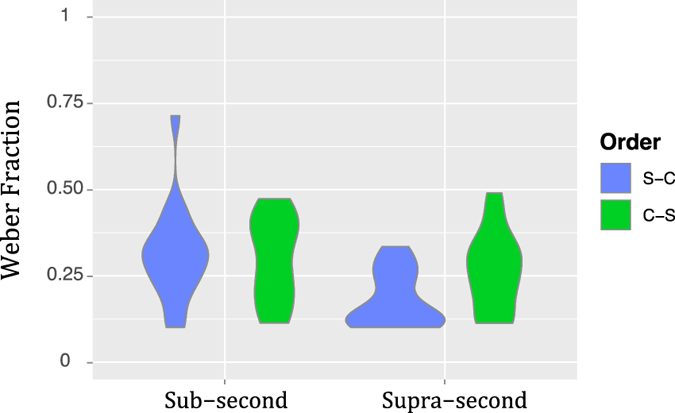
Weber Fractions. Violin plots of the Weber Fractions for each duration range and presentation order, across participants of Experiment 3 (logarithmically spaced ratios). The corresponding ANOVA showed a main effect of duration range (*p* < 0.001, η^2^_G_ = 0.12), a main effect of presentation order (*p* < 0.05, η^2^_G_ = 0.02) and a significant interaction (*p* *<* 0.05, η^2^_G_ = 0.04). Holm-Bonferroni corrected post hoc tests showed that the S-C order of the Supra-second condition differed from all others (vs. Sub-second S-C: *p* < 0.001; vs. Sub-second C-S: *p* < 0.01; vs. Supra-second C-S: *p* < 0.01) and that all remaining comparisons were not statistically significant.

The more symmetrical gradients obtained using logarithmically spaced ratios allowed us to compute and compare WFs. We first tested that the PSEs obtained for this analysis did not differ from the PSEs obtained in the TOE analysis. Wilcoxon tests proved that they were not significantly different (V = 1024, *p* = 0.1). We then proceeded to the WF analysis (Fig. 10), where the rm-ANOVA showed a main effect of Duration (*F*_1,17_ = 15.87, *p* < 0.001, η^2^_G_ = 0.12), with higher values in the *Sub-second* condition; a main effect of Order (*F*_1,17_ = 5.34, *p* < 0.05, η^2^_G_ = 0.02), with higher values in the C-S order; and a significant Duration x Order interaction (*F*_1,17_ = 7.25, *p* < 0.05, η^2^_G_ = 0.04). Holm-Bonferroni corrected *post hoc* tests revealed that the WFs of the S-C order of the Supra-second condition differed from all others (vs. Sub-second S-C: *p* < 0.001; vs. Sub-second C-S: *p* < 0.01; vs. Supra-second C-S: *p* < 0.01). All remaining comparisons were not statistically significant (Sub-second S-C vs. Sub-second C-S: *p* = 0.63; Sub-second S-C vs. Supra-second C-S: *p* = 0.24; Sub-second C-S vs. Supra-second C-S: *p* = 0.52).

## Discussion

The first aim of our study was to assess whether performance on the ETG task is sensitive to presentation order effects. We showed that TOEs appeared in the two duration ranges under consideration, and that this effect held for both linear and logarithmic spacing. Interestingly, effect sizes between Experiments 2 and 3 were inverted. In the linearly spaced experiment, the duration range showed a large effect (η^2^_G_ = 0.48) and presentation order a medium one (η^2^_G_ = 0.14), while the use of logarithmically spaced proportions showed a large effect for presentation order (η^2^_G_ = 0.90) and a medium one for duration range (η^2^_G_ = 0.21). Together with the observed signs of the TOEs, these results reveal different patterns between experiments. In the linearly spaced experiment, subjects overestimated the first sound in the *Sub-second* condition and the second sound in the *Supra-second* range. Instead, when proportions were logarithmically spaced, they overestimated the Comparison duration, with a smaller influence of its position and duration range.

TOEs have been reported in a wide range of tasks^21^ but never in the ETG task. They have mainly been reported when the standard duration was fixed across trials, even though they have been also reported in experiments where both stimuli varied^32, 33^. Having a standard that repeats from trial to trial implies that memory and learning can play a major role in the obtained results and might also introduce other sources of variance. For example, subjects might realize the existence of the standard and try to find it on each trial. Besides, incorrectly identifying the comparison duration as the standard could also lead to distortions in the memory representation of the standard. In our case, standard (and comparison) durations varied from trial to trial, so these factors can be assumed to have had a lower impact.

Our results constitute the first report of TOEs in the ETG task. Presentation order was not taken into account in previous studies^17, 22–24^ and, as our results clearly show, it must be considered when employing the task.

The second aim of our study was to test the symmetry/asymmetry of the temporal generalization gradients while taking presentation order into account, and assessing linear and logarithmically spaced proportions. Experiment 1 showed right asymmetrical gradients in the S-C and C-S orders of the *Sub-second* condition and also in the C-S order of the *Supra-second* range. In the S-C order of the latter the asymmetry emerged in the opposite direction. Experiment 2 showed inverted asymmetries between ranges, that is, right asymmetrical gradients were found in the S-C order of the *Sub-second* condition and in the C-S order of the *Supra-second* range. Conversely, left asymmetrical gradients were found in the C-S order of the *Sub-second* condition and in the S-C order of the *Supra-second* range. Experiment 3 showed that using logarithmically spaced durations yielded more symmetrical gradients but still, results from the S-C order of the *Supra-second* condition were significantly left asymmetrical.

Wearden and colleagues^19^ modelled the *Episodic* version results by modifying the Church and Gibbon model. The original model was created to account for the results of the same task in rats^34^, where the resulting gradient was not asymmetrical. Wearden added the mean of the two durations as normalizing factor, to account for the asymmetry in humans. Thus, the formula for a “yes-equal” response became:

3 $${\rm{abs}}({{t}_{{1}}}^{\ast }-{{t}_{{2}}}^{\ast })/m < {b}^{\ast }$$

where *t*_*1*_ * and *t*_*2*_ * are the two durations to be compared, *m* is their mean (the normalizing factor), and b* is a threshold. The higher the values of *m*, the higher the chances of being below the threshold and giving a “yes-equal” response. This way, it predicts only right asymmetrical gradients, despite the presentation order and duration range of the stimuli. Our results showed left asymmetrical gradients that reflected presentation order effects, therefore contradicting these predictions. Consequently, our findings represent a new empirical constraint calling for a modification of the model.

Our results also have implications for other models of time perception. Apart from Wearden’s proposal, there are other two mainstream models aimed to account for two-interval forced-choice temporal experiments, namely, the Internal Reference Model^35–37^ and the Sensation Weighting Model^21, 32, 38^. Both have been developed for comparative judgements (where subjects have to establish which of the two stimuli was longer), but they have been recently extended to equality judgements^36^. The former was developed for experiments in which the standard was fixed across trials and therefore does not apply in our case. The basic formulation of the Sensation Weighting Model is:

4 $$D={w}_{1}\cdot {X}_{1}-{w}_{2}\cdot {X}_{2}+u$$

where *w*_*1*_ and *w*_*2*_ are the weighting coefficients of the internal representations of the first (*X*_*1*_) and second (*X*_*2*_) stimuli, and *u* is a constant to adjust the mean of *D*. According to this formulation, subjects would judge that durations were equal if *a* < *D* < *b*, where *a* and *b* are thresholds. This account implies that the first and second sounds are weighted differently by the subject and therefore predicts and accounts for the time-order-errors that we observed in our study (see ref. 39). Within this framework, the TOEs arise from the formation of a reference level in the midrange of the stimuli, which is then weighted in the comparisons^38^. This would explain why the TOE was not observed in the *Sub-second* range when using a small number of trials (Experiment 1) and appeared when such number was increased (Experiment 2), as the reference level requires time to be established. In this regard, our results raise the question of whether the *Supra-second* range is more susceptible to this influence.

Previous reports of the Temporal Generalization task^18^ claimed that the fact that temporal generalization gradients superimposed across sub- and supra-second ranges supported Scalar Expectancy Theory, one of the emblematic frameworks assuming the “common timing hypothesis”^40^. We showed that they are different for these two ranges when using the *Episodic* version of the task and taking presentation order into account, due to time-order-errors. However, presentation order effects have been observed in a wide range of tasks, including non-temporal tasks (i.e. weight comparison)^38, 41^. They are considered to be caused by processes beyond the specificity of the temporal domain^21^ and therefore our results should not be interpreted as being in line with the “distinct timing hypothesis”, but rather as the refutation of predictions made by a model that supports the “common timing hypothesis”.

Moreover, the property of superposition has been previously tested via visual inspection or ANOVAs^17, 19^, neither of which is sufficiently robust to such an end. The former is not suitable for hypothesis testing and the latter because of its problems when used on proportional data^25, 26^, as is the case with temporal generalization gradients.

Consequently, the third objective of our study was to compare the WFs of the two ranges. If they were not significantly different, they could be assumed to comply with Weber’s Law. One possible confound in this comparison is that chronometric counting has been shown to improve performance for durations above ~1.18 s^42^ and to reduce the coefficients of variation (as the WF), therefore disrupting the scalar property of variance^43^. Interestingly, our results showed that WFs were smaller only for the S-C presentation order of the *Supra-second* range while not being significantly different in all the remaining comparisons. In other words, WFs were different between duration ranges for one presentation order but not for the other, and they were also different between presentation orders in the *Supra-second* range. If the decrease in the WFs observed in the S-C order of the *Supra-second* range was caused by chronometric counting, it could be expected to have influenced the C-S order in a similar way, which was not the case. Whichever the cause may be, the answer to the question of whether sub- and supra-second timing rely on the same or different mechanisms remains elusive and future studies will be required to elucidate it.

In sum, even though our results do not provide clear evidence in favour or against the scalar property of human timing in the sub- and supra-second duration ranges, they do demonstrate the importance of taking stimulus duration range and presentation order into account. This new constraint should be factored in future studies employing the task and in the models derived from it.

### Limitations

Our study has two main limitations. The first one is that the supra-second condition included, as comparison durations, stimuli that were below the 1s range. Thus, it was not a purely supra-second condition but rather a condition in which the standard duration was supra-second. Future studies could use a pure supra-second condition by choosing a longer standard duration range. The second limitation is that we did not explicitly prevent chronometric counting. We did so to make our conditions comparable. Including a concurrent numerical task within durations of around half a second would have been methodologically incorrect. Not only would that pose higher cognitive demands than if included in a supra-second duration, but it would also be perceptually difficult. It’s worth noting that when chronometric counting was explicitly encouraged in the original version of the task^18^, the resulting psychometric functions were symmetrical when collapsing presentation orders, which was not the case in our study. To overcome this limitation, modifications of the experimental design will be required.

## Conclusions

Our study constitutes the first report of time-order-errors in the ETG task. We also showed differences that arise from the use of sub- and supra-second standards and from linear and logarithmically spaced proportions. In addition, we demonstrated that the current model of the task fails to account for the observed results. Presentation order was not taken into consideration by previous studies and, as our results clearly show, should always be considered. Moreover, we found that the number of trials used influences the observed pattern of results and should therefore also be considered as a crucial factor. Finally, we showed that Weber Fractions also vary as a function of duration range and presentation order. These results afford relevant empirical constraints for future research on the topic.

## Electronic supplementary material

Supplementary Information
