# Supplementary material for: Time-order-errors and duration ranges in the Episodic Temporal Generalization task
Source: Sci Rep. 2017 Jun 1;7:2643. doi: 10.1038/s41598-017-02386-9 (PMC5453992; doi:10.1038/s41598-017-02386-9)
Supplement: Supplementary file 1 — Supplementary Information [file 41598_2017_2386_MOESM1_ESM.pdf]

Time-order-errors and duration ranges in the Episodic Temporal Generalization task.

Ezequiel Mikulan, Manuel Bruzzone, Manuel Serodio, Mariano Sigman, Tristán Bekinschtein, Adolfo García, Lucas Sedeño, Agustín Ibañez

Supplementary Figures

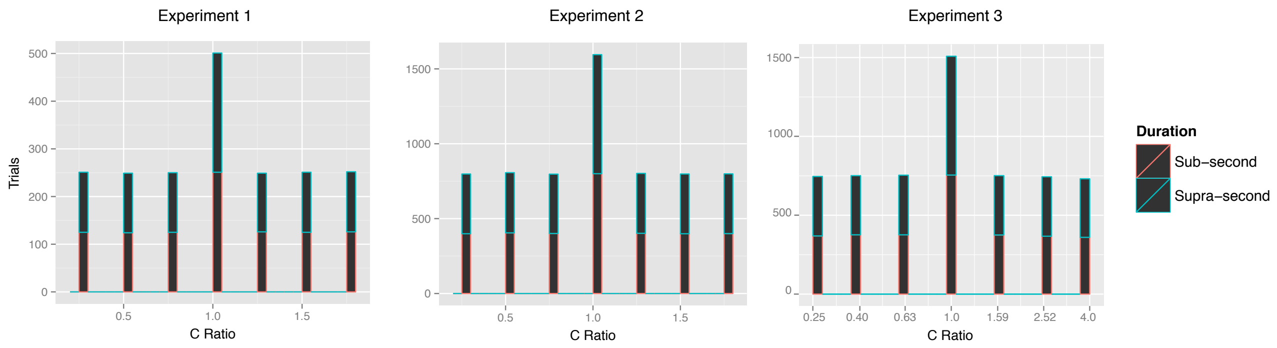

S1. Histogram of trials per condition and ratio.

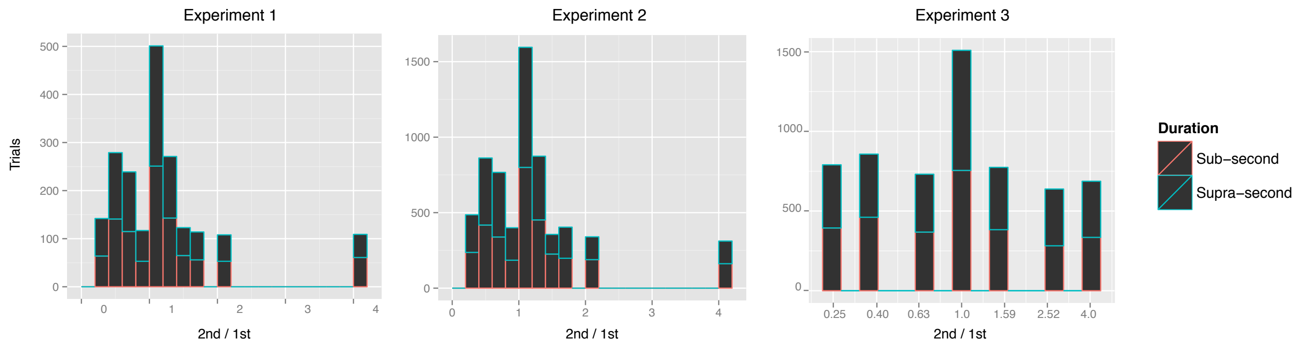

S2. Histogram of trials per condition and 2nd / 1st Proportion.

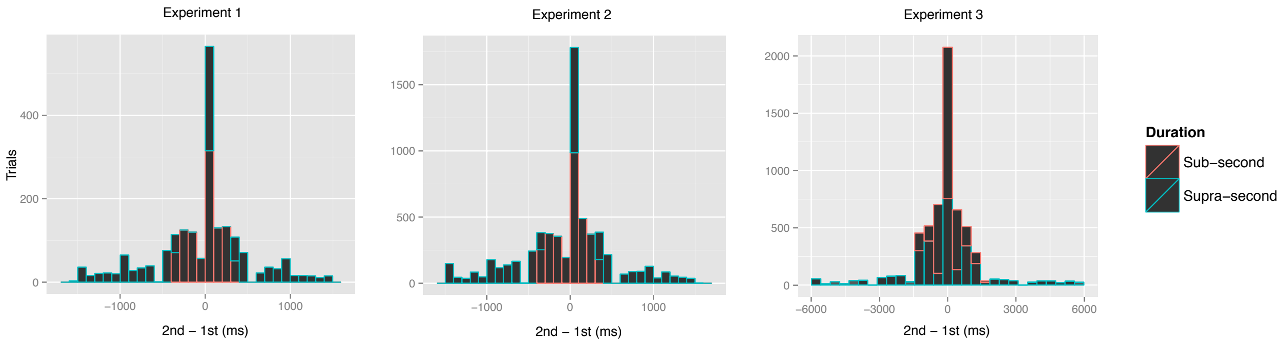

S3. Histogram of trials per duration difference (2nd – 1st).

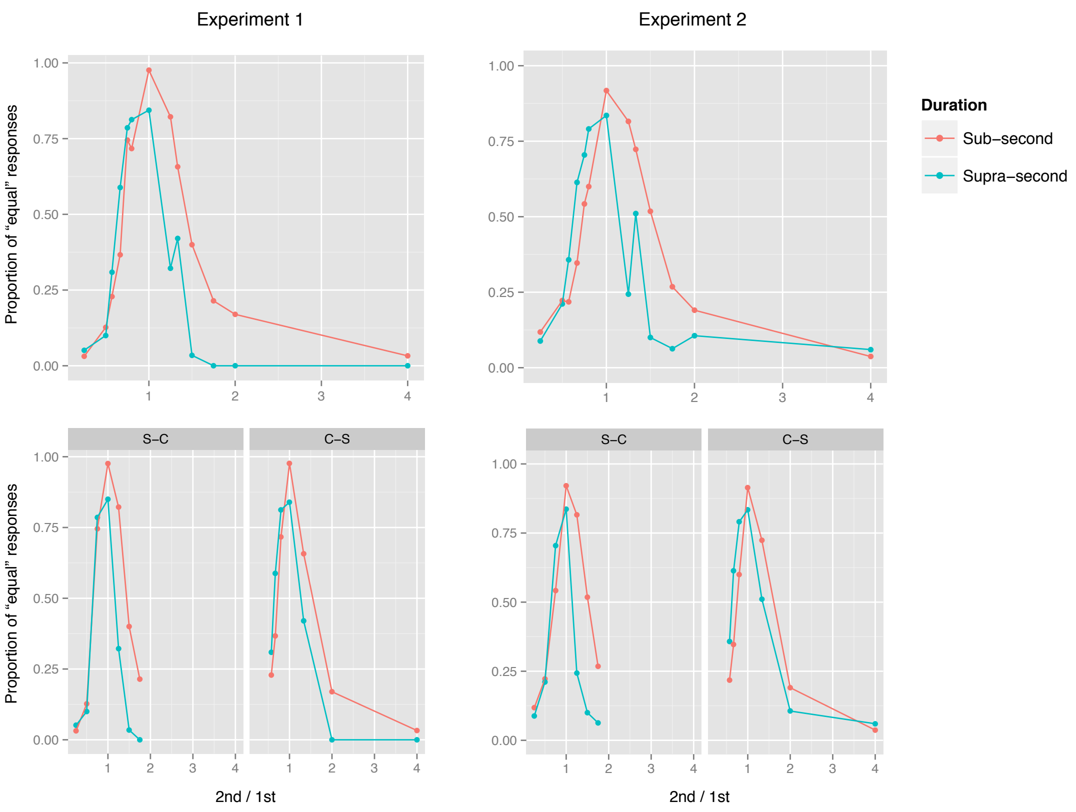

S4. Proportion of “equal” responses per condition and 2nd / 1st proportion of Experiments 1 and 2. Upper panels: collapsing presentation orders. Lower panels: taking presentation order into account.

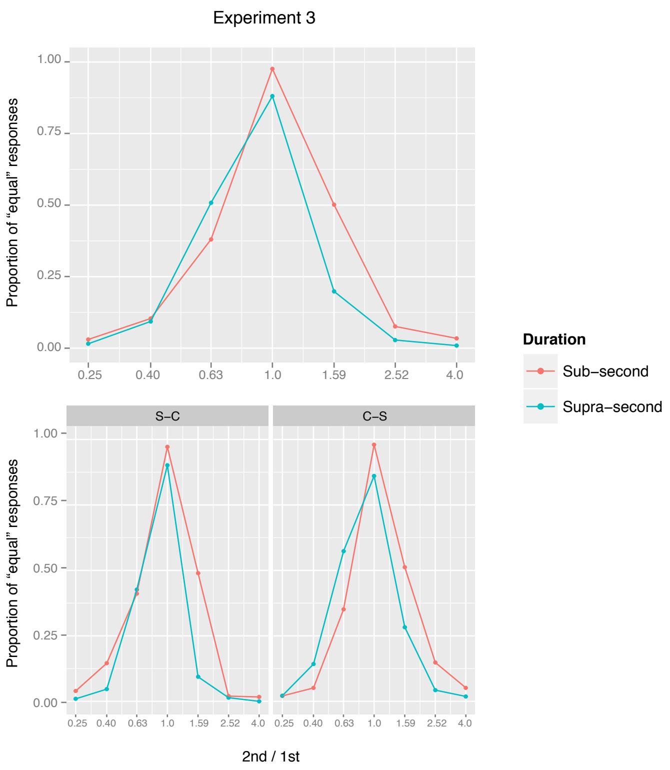

S4. Proportion of “equal” responses per condition and 2nd / 1st proportion of Experiment 3. Upper panels: collapsing presentation orders. Lower panels: taking presentation order into account.
